# Impact Damage Detection in Patch-Repaired CFRP Laminates Using Nonlinear Lamb Waves

**DOI:** 10.3390/s21010219

**Published:** 2020-12-31

**Authors:** Zhenhua Yin, Cheng Li, Ying Tie, Yuechen Duan

**Affiliations:** School of Mechanical and Power Engineering, Zhengzhou University, Science Road 100, Zhengzhou 450001, China; yinzhenhua@gs.zzu.edu.cn (Z.Y.); tieying@zzu.edu.cn (Y.T.); duanyc1984@zzu.edu.cn (Y.D.)

**Keywords:** CFRP laminates, impact behavior, finite element analysis, nonlinear Lamb wave, patch-repair technique

## Abstract

Carbon fiber-reinforced polymer (CFRP) laminates, a key composite material, are widely used in aircraft structures and are susceptible to low-velocity impact (LVI) damage from bird strikes, lightning strikes, hail impacts and other situations. Therefore, finding a method that repairs the damaged structure and detects the effect of these repairs under LVI is a very important goal. In this work, the repair effect of LVI damage in CFRP laminates repaired with patches of various sizes is investigated via experimental and numerical nonlinear Lamb wave analyses. An integrated numerical procedure that combines LVI with nonlinear Lamb wave detection is developed to predict the nonlinear Lamb wave behavior in LVI-damaged patch-repaired CFRP laminates. The CFRP laminate damage in the nonlinear Lamb wave simulation is evaluated based on relative acoustic nonlinearity parameters (RANPs). As a result, the integrated numerical procedure is validated with drop-weight impact tests and RAM-5000 SNAP nonlinear ultrasonic detection system. An optimal patch design is established via interpolation to optimize the absorbed energy, delamination surface area, second RANP and third RANP with different patch repair sizes. These parameters exhibit consistent curve fitting trends, indicating that they can be used as important indicators of impact damage. The optimal circular patch design with a radius of 2.5 r has better impact resistance behavior and repair performance.

## 1. Introduction

Carbon fiber-reinforced polymer (CFRP) laminates are key materials for the development of science and technology in aerospace, national defense, biomedical and other high-tech fields due to their excellent performance [1,2]. One of the critical characteristics of CFRP laminates is their unusually high anisotropy [3]. With the extensive use of composite materials in aircraft structures, many impact-related accidents will inevitably occur, such as bird strikes, lightning strikes, hail impacts and other situations. These impact energies are small but result in barely visible impact damage (BVID), posing a substantial potential threat to the safety of aircraft composite structures [4]. If low-velocity impact (LVI) damage is not identified and addressed, the consequences will be catastrophic. To satisfy the production requirements and reduce the maintenance costs, especially for large and expensive equipment, composite structures that have sustained small impact damage are usually repaired rather than replaced [5,6,7]. Therefore, there is an urgent need to develop patch repair technology to overcome this engineering problem and improve the mechanical properties while minimizing the cost. Because repair techniques that involve external patches are relatively convenient, economic and feasible, they have been extensively applied in industrial production and military application fields. In practical engineering, due to the space and damage location (e.g., skin) limitations of composite aircraft and other equipment, single-sided repair is often used. Generally, in daily routine maintenance and within their service lives, CFRP laminates repaired with external patches are still vulnerable to LVI damage from other foreign objects. To improve the current design methods, researchers have mainly focused on structural health monitoring (SHM) systems, with the aim of ultrasonic nondestructive testing (NDT) and monitoring the presence of damage in a structure during operation, which can effectively increase the safety and reliability of the composite structure [8]. The SHM method based on nonlinear Lamb waves is currently considered to be a promising method for use in the aviation field [9].

Research on the interaction relationship between nonlinear Lamb wave propagation and impact damage in CFRP laminate structures is the basis of damage identification, which has significance. The frequency dispersion of CFRP laminates is substantial, which prevents the use of conventional nondestructive detection methods for SHM in CFRP structures. In recent years, nonlinear ultrasonic techniques have been widely used in early-stage microdamage detection, such as higher harmonic response, subharmonic response, mixed frequency response and nonlinear resonance [10,11]. Several studies have demonstrated that compared with linear features, nonlinear ultrasonic nondestructive detection and evaluation methods mainly capitalize on the frequency-domain signal characteristics and are capable of defect discrimination, thereby overcoming the limitations of traditional ultrasonic methods [12,13,14,15]. Moreover, Lamb waves are almost nondispersive in anisotropic media, so different harmonics can effectively interact during wave propagation.

Several recent studies [16,17] have focused on second harmonic generation in nonlinear Lamb wave propagation and have been implemented in theoretical and experimental microdamage assessments. Rauter et al. [18] employed cumulative second harmonic modes to investigate the impact damage of carbon fiber epoxy composites. Furthermore, they showed that nonlinear acoustic responses are more effective than linear responses for the evaluation of impact-induced microdamage at an early stage and that relative acoustic nonlinearity parameters (RANPs) are reliable and very sensitive indicators of microstructural damage. Hong et al. [19] constructed a probabilistic numerical model to assess the statistical distribution of RANPs based on the BVID in composites. In their study, Lamb waves from healthy and damaged carbon fiber/epoxy laminates were acquired and processed using piezoelectric wafers, and the results from their numerical models were in good agreement with the experimentally obtained statistical distributions of the RANPs. Li et al. [20] investigated the feasibility of assessing LVI damage in CFRP laminates by combining guided wave mixing technology and the mixing frequency peak count method. In their experimental observations of the combined harmonics at the generated mixing frequency, they confirmed the existence of material nonlinearity in laminates. Although some of the existing studies have mainly focused on the LVI behavior of patch repair laminates [6,7,21] or the application of nonlinear Lamb waves for artificial damage detection [22] in composite materials, this modeling method cannot accurately show the interaction between the impact damage and nonlinear Lamb waves. Few studies have focused on the assessment of BVID in patch-repair CFRP laminates under LVI using a higher harmonic propagation response.

In this study, a water jet cutter is used to manufacture circular holes—a damage source—in CFRP laminates. These damaged specimens are repaired through the use of various individual patches of different sizes, which are bonded to one side of the damaged laminates by adhesive. The influences of the CFRP laminates repaired with variously sized patches on the LVI behavior and nonlinear Lamb wave propagation are examined by experimental and numerical methods. The rest of this paper is organized as follows: Section 2 briefly introduces the selection of the frequency and the calculation of the ultrasonic nonlinearity parameter. Section 3 presents the experimental study, including the drop-weight impact test and nonlinear Lamb wave detection. Section 4 proposes an accurate numerical procedure used to predict the nonlinear Lamb wave propagation in LVI damage patch-repaired CFRP laminates, including the LVI step, vibration damping step and nonlinear Lamb wave detection step. Section 5 validates the finite element (FE) modeling procedure and introduces the influence of patches of different sizes on LVI damage and nonlinear Lamb waves as well as the effectiveness of the vibration damping step. Finally, the optimal design is presented and the paper ends with a conclusion.

## 2. Propagation of Nonlinear Lamb Waves

### 2.1. Selection of Frequency

**Figure 1:**
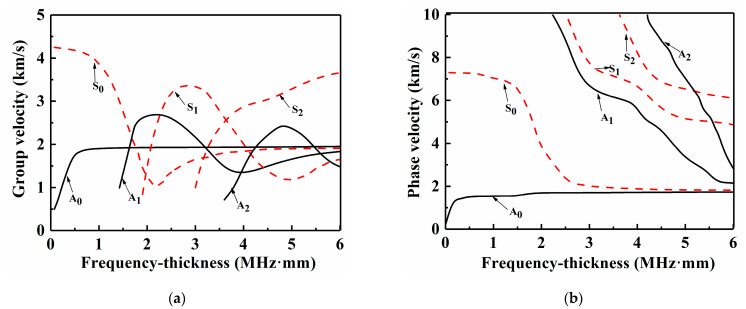
Dispersion curves of the 3.6 mm-thick (0/90)_2s_ carbon fiber-reinforced polymer (CFRP) laminate: (**a**) group velocity and (**b**) phase velocity.

Lamb waves are often utilized in the acoustic guided wave modes of thin laminate structures, wherein the wavelengths of Lamb waves have the same magnitude as the plate thickness. Lamb wave propagation has dispersive, multimodal characteristics [23]. The dispersive characteristics of Lamb waves can be directly described by a dispersion curve, which depicts the relationship between the group velocity or phase velocity in Lamb waves and the frequency-thickness product. The Lamb wave modes are mainly composed of symmetric modes (S_0_, S_1_, S_2_…) and anti-symmetric modes (A_0_, A_1_, A_2_…) based on the phase relationship of the particle vibration in the laminate. Figure 1 represents the group velocity and phase velocity dispersion curves for a 3.6 mm-thick layer CFRP laminate with an orthotropic layup [0/90]_2s_.

CFRP laminates have serious frequency dispersion due to their anisotropy characteristics. When dealing with discontinuous media damage (e.g., delamination and cracks), the received signal usually has more than two modes. The greater the number of modes is, the greater the energy dissipated in the Lamb wave propagation process, and the energy attenuation of low-frequency modes is less than that of high-frequency modes. Many studies [24,25] have highlighted that the fundamental-frequency and double-frequency phase velocities in Lamb waves must be equal to obtain a more obvious second harmonic. The higher the frequency is, the faster the attenuation when propagating in the specimen, which is not conducive to long-distance detection. As the frequency-thickness product increases, the possibility of multimodal Lamb wave excitation in the plate increases, which means that a lower-frequency sensor should be selected for Lamb wave detection of large thickness plates. Due to the limited test conditions, the center frequency of the smallest transducer in the laboratory is 0.5 MHz. Thus, the authors conducted experiments at all optimal frequencies ranging from 0.2–0.6 MHz were considered, and the results showed that when the excitation frequency was selected as 0.5 MHz, and a 1 MHz broadband transducer receives at the same time, the observed higher harmonic signals are more obvious. Moreover, in the A_0_ mode, the phase velocity with frequencies of 0.5–1.5 MHz (frequency-thickness product values from 1.8–5.4 MHz·mm) are also equal. Therefore, the excitation frequency of 0.5 MHz was chosen in this study.

### 2.2. Calculation of the Ultrasonic Nonlinearity Parameter

The ultrasonic Lamb waves are distorted due to the nonlinear interaction with the composite material during propagation, which may generate high-order harmonic components. The second harmonic-based nonlinearity coefficient is a material-dependent property. In the case of a small strain, when the attenuation can be negligible, the approximate solution of the nonlinear wave equation in one dimension in an isotropic solid is obtained by the following progressive analysis method [26,27]:

(1) $$\beta =\frac{8}{k^{2}x}\frac{A_{2}}{A_{1}^{2}}$$

where A_1_ represents the maximum amplitude at ω, A_2_ represents the maximum amplitude at 2*ω*, k=ω/ν (*k* represents the wavenumber, *ω* denotes the excitation signal frequency and *v* is the ultrasonic phase velocity), and *x* represents the signal propagation displacement. *β* is the absolute nonlinear Lamb wave coefficient.

Note that the relevant experimental conditions, for example, the testing device, frequency and sample thickness, are equivalent. Hence, *k* and *x* should be kept constant. Therefore, the following simplified form of Equation (1) can be used to quantify the nonlinear phenomenon:

(2) $$\beta^{\prime }\propto \frac{A_{2}}{A_{1}^{2}}$$

*β*′ is used as the second RANP in the following discussion.

The third RANP can be similarly obtained as follows:

(3) $$\delta^{\prime }\propto \frac{A_{3}}{A_{1}^{3}}$$

where A_3_ is the maximum amplitude at 3*ω*.

The LVI damage of patch-repaired CFRP laminates can be evaluated according to *β*′ and δ′, which are calculated based on A_1_, A_2_, and A_3_ of the frequency-domain spectral signals.

## 3. Experimental Study

### 3.1. Specimen Preparation

The materials used in the experiment were T300/7901 carbon fiber/epoxy prepregs (Weihai Guangwei Composites Co., Ltd., Weihai, China). A circular hole with a radius r of 3 mm was cut into each laminate using a high-pressure water jet cutter (Anhui Aoyu CNC Technology Co., Ltd., Chuzhou, China) to represent a damage source. Each CFRP laminate was composed of eight layers, with a total thickness of 3.6 mm and cross-sectional dimensions of 200 × 100 mm^2^. The layer stacking sequence was [0/90]_2s_. Table 1 shows the mechanical material properties of the CFRP laminates. Since previous studies [7,28] verified that the optimal patch shape for improving the mechanical properties of a damaged CFRP laminate containing a hole is circular, the damaged laminates in this study were repaired with individual external circular patches. The repaired patches were constructed of the same CFRP prepregs as the laminates, but the stacking sequences were [0/90/0], and the total thickness was 0.45 mm. The patches and the damaged CFRP laminates were bonded with a 0.1 mm-thick adhesive film (LJM-200). Moreover, the adhesive film had a Young’s modulus of 3.4 GPa, an ultimate strength of 40 MPa, and a Poisson’s ratio of 0.35. The distance from the damage hole center to the CFRP laminate center was set to 20 mm [29].

**Figure 2:**
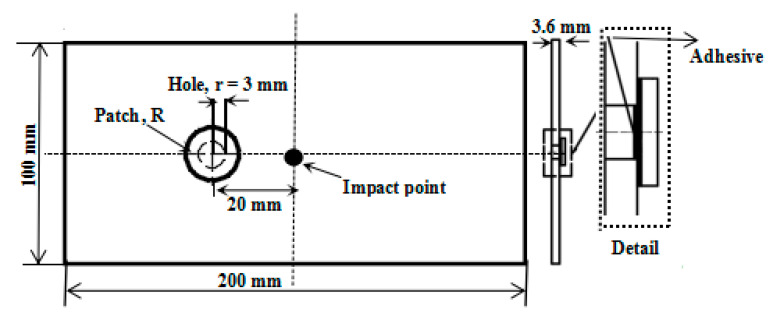
Geometric configuration of the repaired plates.

Figure 2 shows the geometric configuration of the repaired plates. In the experimental study, drop-weight impact tests were carried out on damaged CFRP laminates that were repaired using eight circular patches with radii of 0 r (namely, no patch), 1.5 r, 1.75 r, 2 r, 2.25 r, 2.5 r, 2.75 r, and 3 r (r: hole radius). At least three specimens of each group were prepared for testing to ensure that each repaired laminate obtained effective experimental results.

### 3.2. Drop-Weight Impact Test

**Figure 3:**
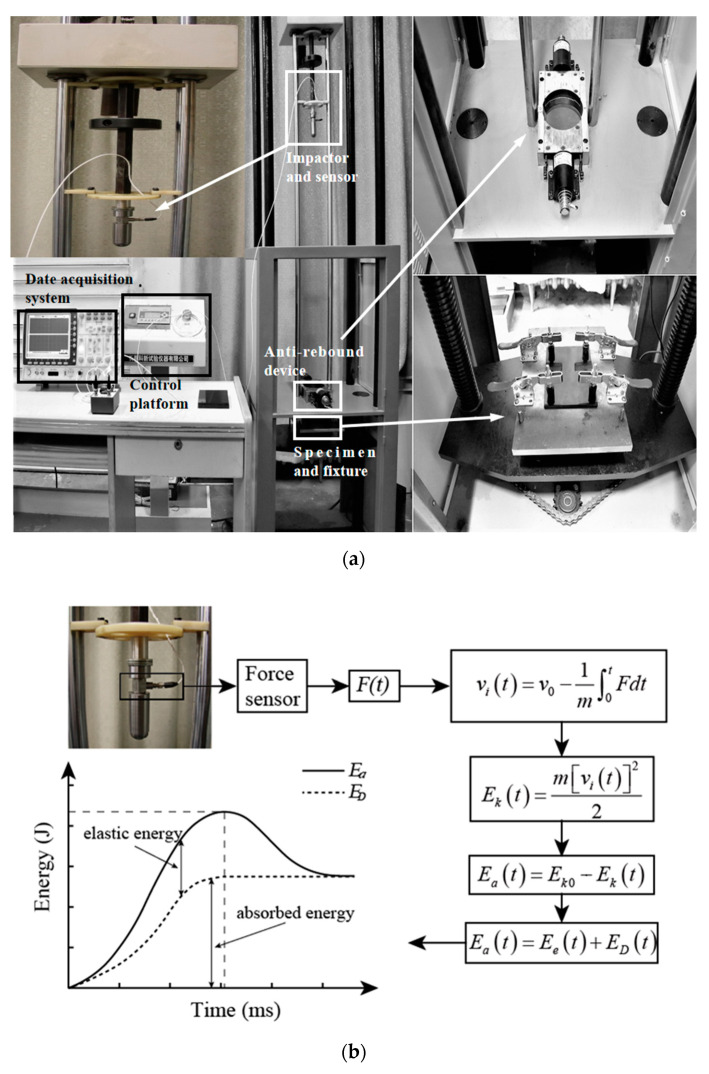
Low-velocity impact (LVI) test: (**a**) photograph of the system and (**b**) scheme of the experimental calculation.

A drop-weight impact experiment was carried out in accordance with American Society for Testing and Materials (ASTM) D7136/D7136-07 [30]. The LVI experiments were performed with an XBL-300 drop-weight impact testing machine (Changchun Kexin Test Instrument Co., Ltd., Changchun, China). Figure 3a shows the impact testing system, which included an impactor, a sensor, an anti-rebound device, a data acquisition system and a specimen clamp. The repaired CFRP laminate was placed on a support fixture with a rectangular cutout of 125 × 75 mm^2^, which enabled the impactor to conduct an impact test on the laminate without interference. Additionally, prior to the impact test, four rubber clamps were applied to fix the laminate on the support fixture to ensure stability during the impact process.

A hemispherical impactor with a radius of 12.5 mm and a weight of 2.5 kg was placed at a height of 0.65 m to perform the LVI tests of the repaired CFRP laminate. It was presumed that no other energy loss occurred because of friction during the impactor falling process. Thus, the initial LVI velocity and initial energy were calculated as 3.58 m/s and 16.0 J, respectively.

After the LVI test, the impact force *F* and time *t* data of the impactor were obtained by a 208C03 universal ICP force sensor from the American PCB Piezoelectric Company, and the corresponding impact velocity at any time point could be obtained by the impactor mass. The LVI process usually takes only a few milliseconds. It was assumed that the energy lost by the impactor due to impact was completely absorbed by the CFRP laminate, so the change amount in the kinetic energy *E_k_(t)* of the impactor was equal to the energy *E_a_(t)* absorbed by the CFRP laminate. However, the *E_a_(t)* absorbed by the laminate caused elastic energy *E_e_(t)* and irreversible energy *E_D_(t)* to be consumed by the laminate due to local damage.

The energy absorbed is the essential reason for the LVI damage of the CFRP laminates. The specific test calculation flowchart is shown in Figure 3b, where *v*_0_ and *v*_i_(*t*) are the initial and current velocities of the impactor, respectively; *m* represents the impactor weight; *t* represents the duration time from the beginning of the impact contract to the end of the impact contact; and *F*_i_(*t*) represents the experimental impact force at time t. Under the same conditions, three identical representative specimens of each size repair were tested, and the variation of their measured impact energy curves was approximately 1.5% to ensure the reliability of the results.

### 3.3. Nonlinear Lamb Wave Detection

The experiment adopted the sensor arrangement method for the transmitting and receiving components to measure A_1_, A_2_, and A_3_. To minimize the generation of harmonics, the transmitted signal should not overlap with the received signal during propagation. Therefore, we used sine pulse excitation in the experiment and recorded the received time-domain signal with an oscilloscope. Moreover, a Hanning window was added for debugging, and the oscilloscope waveform was observed to ensure that the measured samples generated a single-mode Lamb wave.

**Figure 4:**
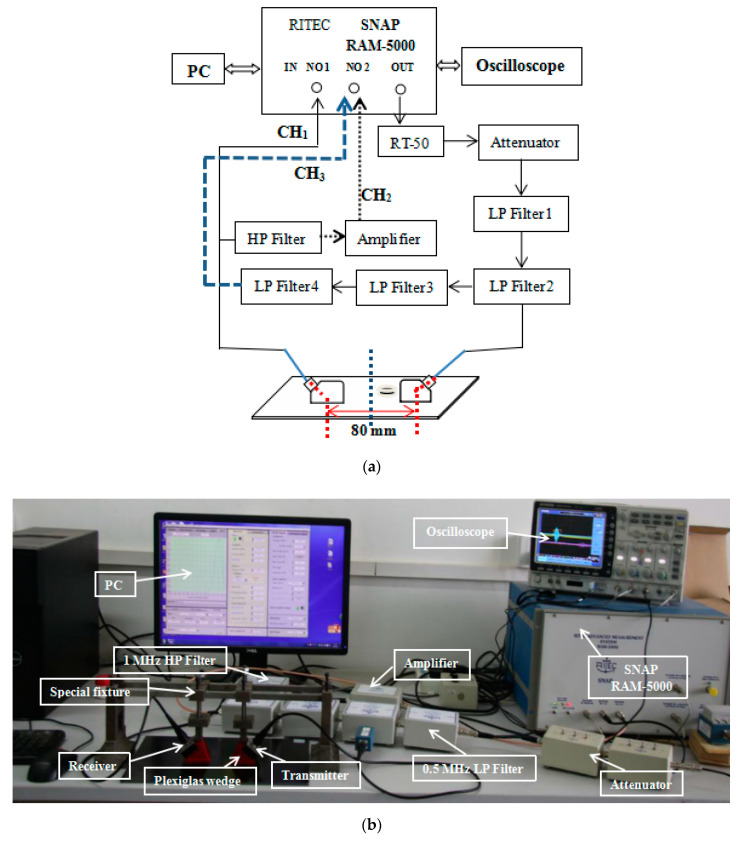
Nonlinear ultrasonic detection system: (**a**) block diagram of the experimental setup and (**b**) photograph of the system.

The experimental setup of nonlinear ultrasonic detection is shown in Figure 4. The key component of this setup, the computer-controlled RITEC RAM-5000 SNAP ultrasonic detection system, generated high-power tone burst signals and detected nonlinear Lamb waves. In this experiment, two longitudinal transducers with central frequencies of 0.5 MHz (Olympus NDT, A414S) and 1 MHz (Olympus NDT, A407S) were employed as the transmitter and the receiver, respectively, and mounted on Plexiglas wedges at an angle of 45°. The optimal distance between the transducers was set to 80 mm. Note that 7501 high-vacuum silicone grease was utilized as the coupling agent, and the excitation transducer and receiving transducer were positioned along the same straight line. A special fixture was employed to ensure full coupling between the transducers and the samples during detection under constant pressure. The system emitted a sinusoidal pulse signal with nine cycles at a frequency of 0.5 MHz. After a 0.5 MHz low-frequency filter group removed the high-frequency interference generated by the device, the 0.5 MHz transducer generated ultrasonic Lamb waves that entered the samples. The 1 MHz broadband transducer received the propagated excitation signal, which was one of the fundamental waves (CH_1_) and the second harmonic channels (CH_2_). After receiving the signal at CH_2_, which passed through a 1 MHz high-pass filter and underwent 30 dB preamplification, the RAM-5000 SNAP system performed signal extraction and processing. The attenuator was used to attenuate the third harmonic (CH_3_), which was attributed to spontaneous self-receiving, by 18 dB.

The nonlinear detection system was used to measure the frequency domain signal response of the sample under the same conditions three times, and the average values of A_1_, A_2_, and A_3_ were taken. To overcome the influence of random noise, the relative error of the repeated measurement was set below 2%.

## 4. Finite Element Simulation

**Figure 5:**
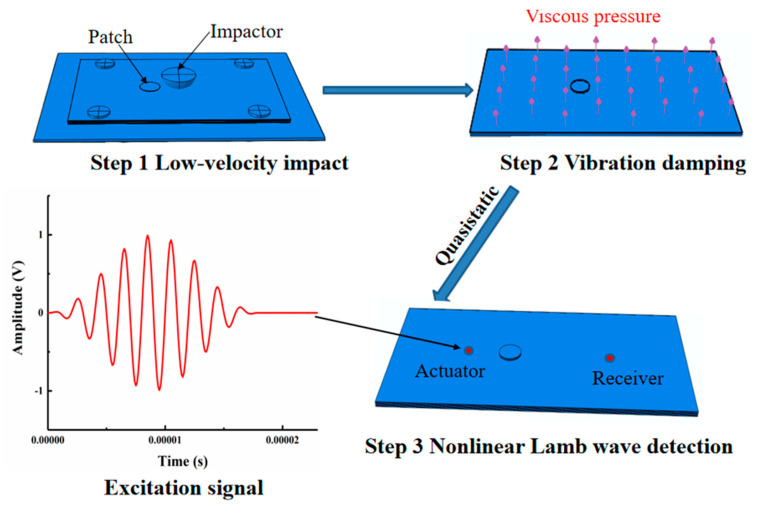
Finite element (FE) simulation flowchart.

This section introduces an accurate FE model employed to predict the nonlinear Lamb wave behavior in LVI-damaged CFRP laminates that were repaired with patches of various sizes. In the past, most domestic and international researchers have simulated the damage evolution and mechanical responses of composite materials only during impact or with preset damage, such as cracks or delamination, when investigating Lamb wave propagation in damaged structures, resulting in deviations of the results. In this paper, an improved FE modeling method was used to predict the LVI damage process of repaired CFRP laminates—producing nonartificial damage—and to detect the induced damage. This work combined the generation of LVI damage and Lamb wave behavior within one model and explored the interaction between LVI damage and the nonlinear Lamb wave response. The FE model flowchart, which contains three steps, is shown in Figure 5. The impactor collided with the repaired specimens under certain conditions to cause LVI damage, and then the model reached quasistatic equilibrium within a minimal number of increments under viscous pressure. After a short time delay, the Lamb wave excitation signal was loaded for the nonlinear detection simulation of postimpact damage.

### 4.1. Low-Velocity Impact Step

**Figure 6:**
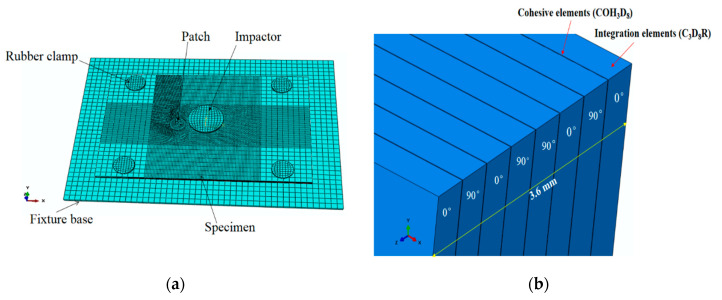
Diagram of the FE model for the LVI test: (**a**) specimen repaired containing a circular patch and (**b**) the layup of the CFRP laminate.

To further study the impact damage characteristics of unrepaired laminates and laminates repaired with single patches of different sizes, a meshed simulation model for CFRP laminates repaired with a circular patch of radius 2.5 r, as illustrated in Figure 6, was developed with Abaqus/Explicit [31] software. The FE model was validated by considering the drop-weight impact test cases (Section 3.2) with a three-ply circular patch and stacking sequence [0/90/0]. C3D8R solid elements were utilized to mesh the specimen, circular patch, impactor, fixture base and rubber clamps. Moreover, COH3D8 cohesive elements were also placed between each layer of the CFRP laminate. The patch material was consistent with that of the laminate, and the adhesive layer between the damaged CFRP laminate and the patch was also simulated by COH3D8 elements, where the thickness of the adhesive layer was taken as 0.1 mm [32,33]. The initial velocity of the impactor in the U3 direction was 3.58 m/s. Accordingly, simulated drop-weight impact tests were conducted on CFRP laminates repaired with radii ranging from 1.5 r to 3.5 r to determine the optimal patch size design parameter.

#### 4.1.1. 3D Hashin Progressive Failure Criteria

Generally, the LVI damage of CFRP laminates consists of two main categories: intralaminar damage and interlaminar damage. Intralaminar damage results in fiber breakage and matrix cracking, whereas interlaminar impact damage is mainly delamination and debonding. The three-dimensional (3D) Hashin damage criteria [34,35] and damage evolution are effectively applied in many numerical simulations to predict the intralaminar damage initiation and progressive failure process of CFRP laminates that were repaired with patches of various sizes. A continuum damage model (CDM) was utilized to calculate the residual strength of the damaged laminates in this study. In addition, to assess the LVI damage of CFRP laminates, a user material subroutine (VUMAT) was applied to implement the 3D Hashin failure criteria.

#### 4.1.2. Cohesive Zone Model

The cohesive zone model (CZM) [36] was selected for simulating the delamination damage that occurs between the different layers of CFRP laminates, along with the debonding between the patches of various sizes and the CFRP laminates containing holes. This model was able to accurately simulate the delamination damage when compared with the experimental results, which showed agreement with each other. The CZM uses a traction-separation law to determine the onset of interlaminar damage, while predicting the occurrence of adhesive interface damage [37]. The interlaminar damage of the CFRP laminates that were repaired with patches of various sizes was controlled by a quadratic separation law [38], and delamination and debonding damage occurred when the damage variable reached 1. A bilinear response model was used in this study. The CZM approach was also successfully implemented to simulate the interlaminar impact damage behavior of the interaction among the zero-thickness cohesive elements.

### 4.2. Vibration Damping Step

After an LVI, even if the impact simulation has been completed, the CFRP laminate continues to vibrate for a long time. To prevent this vibration from affecting the received Lamb wave signal, it was necessary to dampen all the vibrations caused by the impact. If no vibration damping step was utilized in the FE model, Lamb wave propagation would occur on the vibrating laminate, in which the vibration amplitude would be larger than the amplitude produced by the actuator. Therefore, the vibrations in the CFRP laminate can be dampened before Lamb wave detection.

Viscous pressure loads [39] allow the laminate to achieve quasistatic equilibrium in the smallest incremental steps and are defined by Equation (4), as follows:

(4) $$p=-c_{v}vn$$

where *p* denotes the viscous pressure, *c_v_* represents the viscous pressure coefficient, ***v*** represents the velocity vector at a point on the laminate surface, and *n* represents the unit outward normal vector to the surface.

### 4.3. Nonlinear Lamb Wave Detection Step

For nonlinear dynamic problems, such as the propagation of nonlinear Lamb waves, explicit dynamic module analyses are adopted to calculate the model. According to the physical characteristics of Lamb wave propagation, to ensure the correctness of the numerical solution, the model required a sufficiently small cell mesh size and time step. Moreover, each wavelength had at least 10 to 20 cell nodes [40]. The spatial discretization of *Lx*, *Ly* and *Lz* satisfied the following conditions:

(5) $$\frac{\lambda_{min}}{20}\leq \max\left(L_{x},L_{y},L_{z}\right)\leq \frac{\lambda_{min}}{10}$$

where *λ*_min_ is the smallest wavelength and *Lx*, *Ly* and *Lz* are the node spacings in the *x* direction, *y* direction and *z* direction, respectively. Another necessary condition was that the time step had to accord with the following conditions:

(6) $$\Delta t\leq \frac{\min(L_{x},L_{y},L_{z})}{c_{g}}$$

where *c*_g_ represents the group velocity of the Lamb waves. According to the calculation of Equations (5) and (6), to ensure the accuracy between LVI damage and Lamb wave detection, the impact damage and nonlinear Lamb wave detection zone were very densely meshed, and very small elements were utilized to accommodate the complex nonlinear detection behaviors of the composite materials. For the unrepaired CFRP laminate in this study, the mesh size of the impact damage and the nonlinear Lamb wave detection zone were set to 0.5 mm (Figure 6a), and the mesh size in the remainder of the zone was set to 2 mm. The mesh size in the patch zone was set to 0.2 mm. The time step was 1 × 10^−8^ s in this study. This level of refinement has been verified to sufficiently describe LVI damage behavior. 

**Figure 7:**
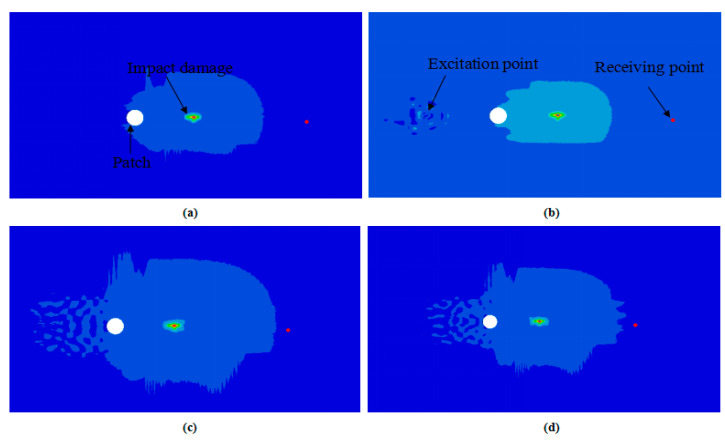
Frame sequence of the nonlinear Lamb wave propagation in LVI damage patch-repair CFRP laminates: (**a**) 0 µs, (**b**) 10 µs, (**c**) 20 µs and (**d**) 35 µs.

The Lamb wave signals were excited by applying an equivalent displacement load to a node (−40, 0, 3.6), and the equivalent displacement load could be obtained by Equation (7), as follows:

(7) $$u\left(x,t\right)=\frac{A}{2}\left[1-\cos(\frac{2\pi tf}{n}\right)]\cdot \sin(2\pi tf)$$

where *x* is the real-time amplitude, *t* is the wave propagation duration, *A* represents the maximum adjustment amplitude, *f* represents the excitation signal frequency (*f* = 0.5 MHz), and *n* is the cycle number (*n* = 9). The excitation point and receiving point were located on either side of the CFRP laminate midpoint, and the distance between them was 80 mm. Figure 7 illustrates the Lamb wave propagation displacement nephogram in the LVI-damaged CFRP laminate that was repaired with a patch of radius 2.5 r at different frame sequences. By considering different acquisition times, the sensors recorded the A_0_ modes. The interplay between the nonlinear Lamb wave propagation and the LVI damage produced scattered waves, which enabled the damage inside the CFRP laminate to be detected.

## 5. Results and Discussion

First, according to the experimental and numerical results, the nonlinear Lamb wave propagation behaviors of the LVI-damaged CFRP laminates that were repaired with patches of various sizes are discussed, and the correctness of the FE model strategy is verified by comparing these results. Finally, the optimal patch size design is determined based on the interpolation method.

### 5.1. Validation of the FE Modeling

**Figure 8:**
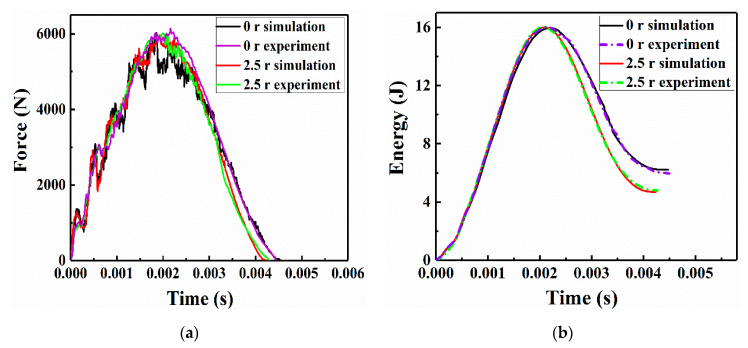
Numerical and experimental results of the specimens repaired with patches of radii 0 r and 2.5 r: (**a**) impact force and (**b**) absorbed energy.

The FE models were established based on the experimental background of the drop-weight impact tests and nonlinear Lamb wave detection presented in Section 3. To verify the correctness of the FE modeling, the simulation and experimental results of the specimens that were repaired using circular patches with radii of 0 r (unrepaired) and 2.5 r were compared. Figure 8 illustrates the numerical and experimental impact force-time and absorbed energy-time historical curves of the patch-repaired CFRP laminates. As shown in Figure 8a, the numerical and experimental maximum forces of the unrepaired specimens were approximately 6020.6 N and 6136.8 N, respectively, whereas those of the specimens repaired with a patch of radius 2.5 r were approximately 5961.4 N and 6012.3 N, respectively. Therefore, the slight differences between the experimental impact force peak values and numerical impact force peak values of these two specimens were less than 1.93% and 0.85%, respectively. Moreover, for both specimens, the time loci that correspond to the impact force peaks in the experimental and numerical cases show agreement. In addition, the impact-absorbed energy curves in Figure 8b show that the absorbed energy and its corresponding time locus were approximately equal in the experimental and numerical cases. Considering the energy dissipation and friction in the LVI test, the deviations between the impact force peaks and absorbed energy were acceptable. It was concluded that the impact process of the unrepaired structure lasts longer, and the impact force and absorbed energy also reach a larger magnitude when compared with the 2.5 r repaired structure. As discussed in the literature [7], the more severe the fluctuation of the impact force, the more impact energy absorbed by the CFRP laminates that were repaired with patches. Thus, the impact energy absorbed by the unrepaired specimens was higher than that of the specimens repaired with a patch with a radius of 2.5 r.

**Figure 9:**
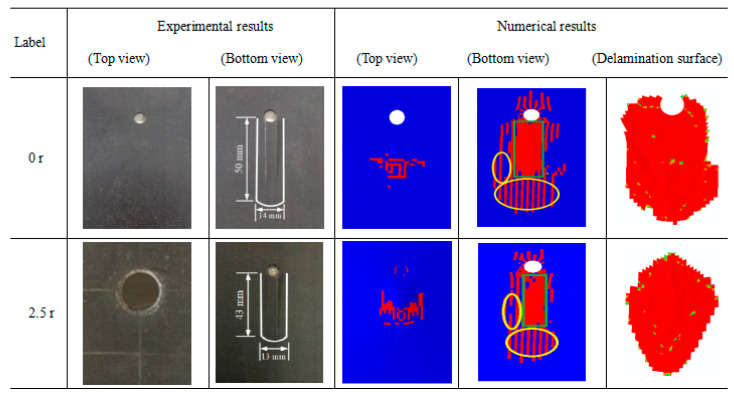
Matrix tensile and delamination surface damage from the experimental and numerical simulations.

Figure 9 shows the matrix tensile damage and delamination surface area of the two specimens. According to the experimental and numerical simulation results, no obvious damage is observed from the top view of the specimen structures. However, the bottom view shows the damage more clearly. Here, the delamination surface area is mainly composed of all the cohesive elements between the different layers in the CFRP laminate deleted during the LVI process. The damaged area was significantly reduced after the specimen was repaired. In addition, according to the numerical results, the bottom damage area of the unrepaired specimen structure was wider than that of the specimen repaired with a patch of radius 2.5 r. In the simulation, the matrix tensile damage corresponding to the yellow circles in Figure 9 was relatively scattered. Because of the discontinuity, experimental damage cannot be observed with the eye. The actual damage sustained during the experiment corresponds to the concentrated area of numerical damage (i.e., the green rectangles in Figure 9). The simulated bottom damage of the two specimen structures expanded along the long axis, which was basically consistent with the experimental results.

The detailed delamination surface areas from the experimental and numerical results of the two repaired specimens are listed in Table 2. In the experimental tests, the surface area values were calculated by the approximate rectangular area formula method. In the FE models, the delamination surface damage values were automatically calculated by a damage envelope area plugin tool. The differences in the experimental and FE simulation results between the two specimens were less than 1.57% and 4.47%, respectively. This further proves that the FE simulation strategy used to study the LVI behavior of CFRP laminates repaired with different patches is reliable.

**Figure 10:**
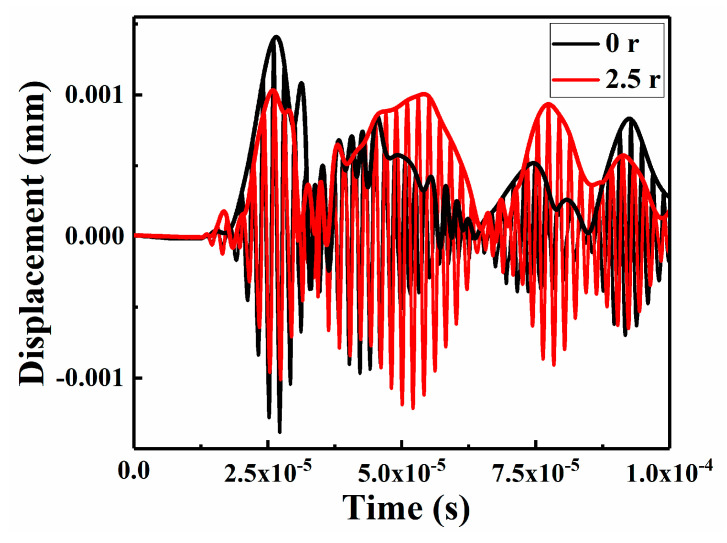
Numerical Lamb wave time-domain signal responses and envelopes.

**Figure 11:**
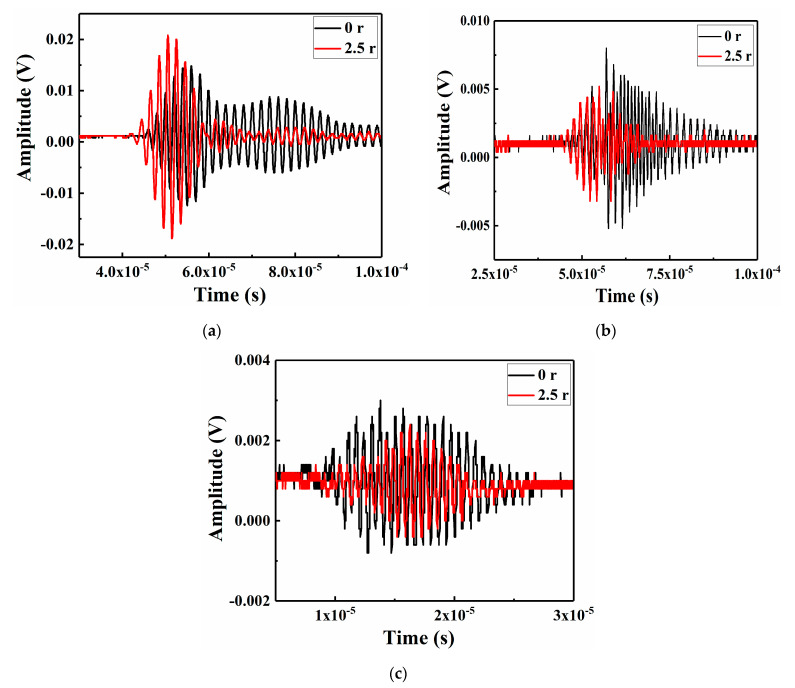
Experimental Lamb wave signal recorded by the sensor in the RITEC system: (**a**) fundamental wave, (**b**) second harmonic and (**c**) third harmonic.

The displacement-time data of the patch-repair CFRP laminates under LVI in the nonlinear Lamb wave detection model were extracted as the received time-domain signal to study the Lamb wave propagation characteristics in the FE simulations. In contrast to the numerical simulation method, the fundamental wave and second harmonic and third harmonic signals were acquired using the RAM-5000 SNAP detection system by different channels in the experimental process. Figure 10 and Figure 11 show the time-domain signal results of the simulation and the experimental value at the receiving point.

**Figure 12:**
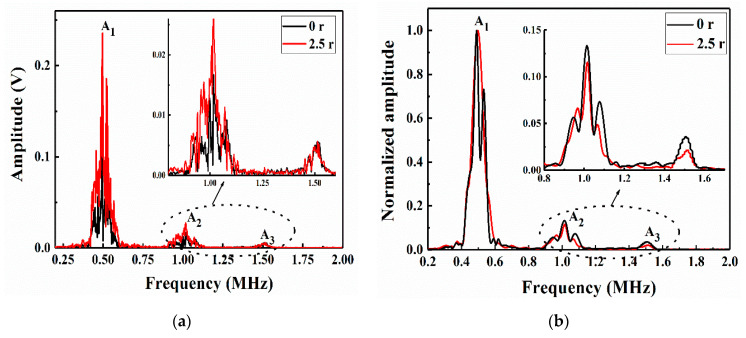
Frequency-domain responses in the numerical model of the unrepaired and repaired CFRP laminates: (**a**) actual amplitude and (**b**) normalized envelope amplitude.

**Figure 13:**
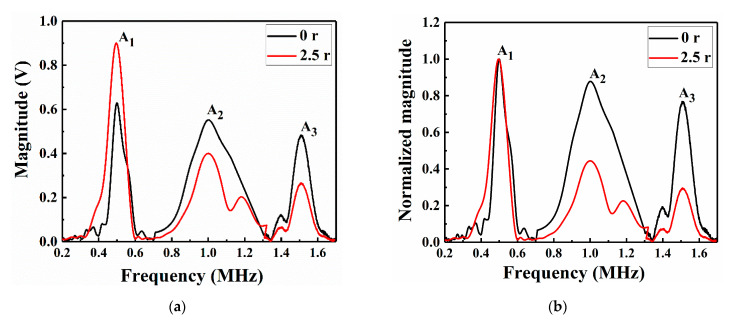
Frequency-domain responses in the experiments with the unrepaired and repaired CFRP laminates: (**a**) actual amplitude and (**b**) normalized amplitude.

The envelope signal amplitude of the specimen repaired with a patch radius of 2.5 r was smaller than that of the unrepaired specimens, which might be due to the reduction in the Lamb wave energy attenuation in the CFRP laminate after repair. To study the nonlinear phenomenon in greater detail, the signals were switched from the time domain to the frequency domain by a fast Fourier transform (FFT), as shown in Figure 12 and Figure 13.

From the numerical and experimental results (Figure 12 and Figure 13), the unrepaired specimens and those repaired with a patch of radius 2.5 r both contained obvious A_1_, A_2_ and A_3_ at frequencies of 0.5, 1 and 1.5 MHz, respectively. To better compare and verify the agreement of the numerical model with the experimental results, a normalization method was used to obtain the amplitudes in the frequency-domain signals Figure 12b and Figure 13b show that the unrepaired specimens had a more obvious second harmonic and third harmonic than those repaired with a patch of radius 2.5 r. This was because the greater the impact damage of the unrepaired structure was, the greater the nonlinear effect of the Lamb wave propagation; thus, the normalized amplitudes of the second and third harmonics were relatively high. This difference in amplitude size can be used to detect LVI damage and evaluate the advantages and disadvantages of the repair effects. The A_1_, A_2_ and A_3_ data in Figure 12a and Figure 13a were extracted, as shown in Table 3. The second RANP (*β*′) and the third RANP (δ′) were obtained by calculating Equations (2) and (3), respectively. The amplitudes in the experimental data were obviously larger than those in the FE simulation data. Moreover, the second RANP and third RANP values of the unrepaired laminates were larger than those of the laminates repaired with a patch of radius 2.5 r in both the numerical simulations and the experiments. This was because the patch-repaired specimens reduced the nonlinear effects of Lamb wave propagation. Due to the influence of the nonlinear Lamb wave detection environmental factors, the error in the nonlinearity coefficient was acceptable within a certain range, indicating that the FE model fit the experiment more accurately. The results showed that the impact damage and the acoustic nonlinearity model of the patch-repaired specimens were effectively established by the FE simulation method. Moreover, the validation of the FE models was also clarified in this study.

### 5.2. Influence of Patch Size on Impact Damage

**Figure 14:**
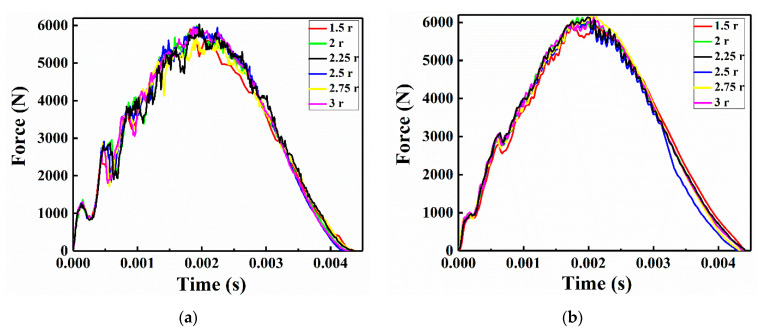
Impact force curves of the CFRP laminates repaired with patches of various sizes: (**a**) numerical and (**b**) experimental.

Since the patches were verified to significantly enhance the mechanical properties of damaged laminates containing holes and reduce the nonlinear effects of Lamb wave propagation, the influence of different patch sizes was studied using numerical and experimental approach. Consequently, LVI tests were carried out on CFRP laminates that were repaired using various circular patches with radii ranging from 1.5 r to 3 r. The predicted and experimental impact force-time curves from these tests are illustrated in Figure 14.

As shown in Figure 14, the impact force curves of the numerical simulation and experimental indicates that fluctuations in the impact force occurred during each impact test and that these fluctuations exhibited similar trends. Additionally, the maximum fluctuation in the impact force appeared in the CFRP laminate that was repaired with a patch of radius 1.5 r. The specimen repaired with a patch of radius 2.5 r exhibited the shortest impact time. Moreover, the other repaired laminates also have similar impact oscillation trends, and the maximum force peak appeared at 2.0 ms.

**Figure 15:**
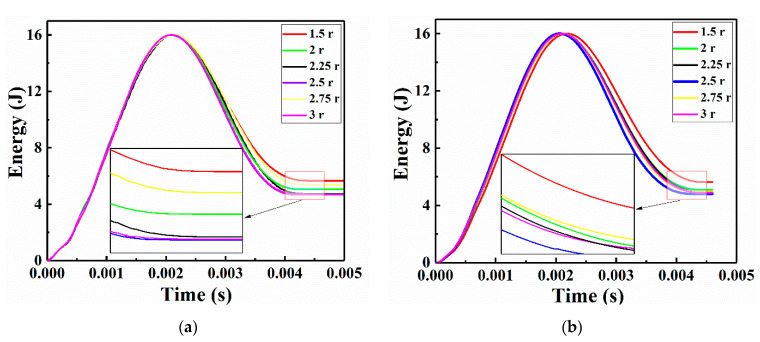
Impact energy curves of the CFRP laminates repaired with patches of various sizes: (**a**) numerical and (**b**) experimental.

The numerical and experimental absorbed energy-time curves are shown in Figure 15, the CFRP laminate that was repaired with a patch of radius 1.5 r absorbed the most impact energy. The specimen repaired with a patch of radius 2.5 r absorbed the least impact energy. The curve differences of the CFRP laminates repaired with patches of various sizes might be due to the penetration depth degrees of the impactor into the samples, as discussed in the literature [7]. The deeper the impactor penetrated the repaired CFRP laminates, the more impact energy was absorbed. The corresponding work has a limitation to further study penetration depth by the current experimental measurements setup. The absorbed energy was an appropriate physical variable for evaluating the repair performance advantages and disadvantages of damaged CFRP laminates.

**Figure 16:**
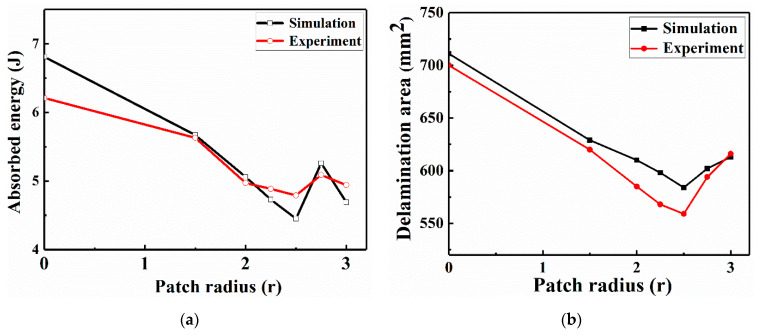
Numerical and experimental curves of the specimens repaired with patches of various sizes: (**a**) absorbed energy and (**b**) delamination area.

The numerical and experimental absorbed energy and delamination area curves decreased with an increase in the patch size and are shown in Figure 16. The delamination area value corresponds to the absorbed energy value. The greater the absorbed energy is, the greater the delamination damage area. When the repaired-patch radius size reached 2.5 r, the corresponding damage degree reached the minimum value after impact. Moreover, the repair performance of CFRP laminates is reflected not only in the plane direction but also in the out-of-plane direction. If the patch sizes exceed the critical radius, the large patch mainly improves the anti-impact behavior in the out-of-plane direction. Thus, the impactor cannot penetrate to the depth of the other specimens, resulting in the repaired laminates having a larger delamination area [7]. Therefore, as the patch radius continues to increase, the absorbed energy and the delamination area tend to increase. The specimen repaired with a patch of radius 2.5 r exhibited better repair performance than the other specimens. In addition, the numerical absorbed energy value of the unrepaired structure was 6.81 J, whereas that of the optimal patch-repaired structure was 4.45 J. The experimental absorbed energy value of the unrepaired structure was 6.21 J, whereas that of the optimal patch-repaired structure was 4.79 J. The predicted and experimental optimal patch design can significantly increase the impact resistance performance of the CFRP laminate that contains a hole by approximately 34.65% and 22.87%, respectively. Therefore, the mechanical properties of damaged CFRP laminate structures can be effectively restored by using appropriate parameters for the external patch-repair operation.

### 5.3. Effectiveness of the Vibration Damping Step

**Figure 17:**
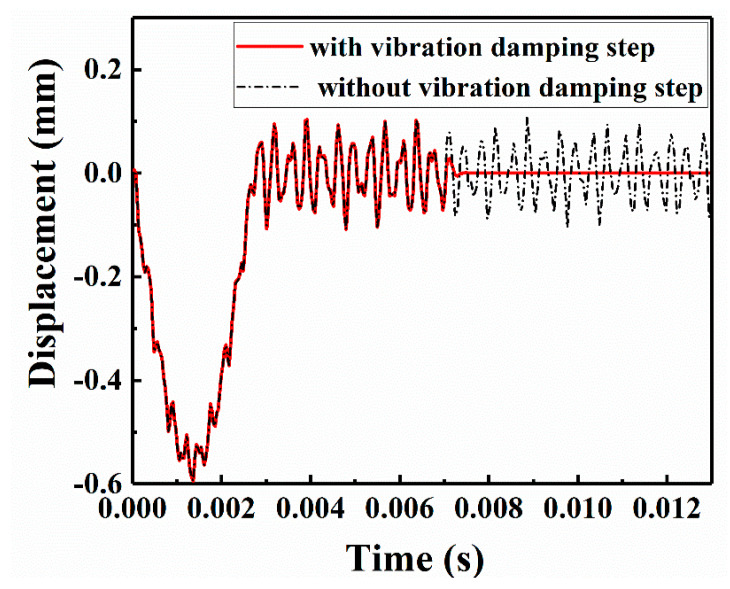
Effectiveness of the vibration damping step on the upper surface of the repaired CFRP laminate.

To verify the effectiveness of the vibration damping step, two analysis steps in the FE models must be established for comparison: one with a vibration damping step and one without a vibration damping step. The displacement-time curves of the central node (x = 40, y = 0, z = 3.6; the coordinate origin of the plate center) on the upper surface of the specimen repaired with a patch of radius 2.5 r are illustrated in Figure 17. The total time of the extracted data was 0.013 s, and the first 0.007 s was the impact analysis step. The black curve in the figure represents the model without the vibration damping step, and the point continued vibrating at a high amplitude until 0.013 s. However, after the vibration damping step was added, the laminate quickly reached a quasistatic equilibrium state after 0.007 s (i.e., the red solid line). This analysis clearly highlighted the necessity and feasibility of the vibration damping step for receiving correct Lamb wave response signals.

**Figure 18:**
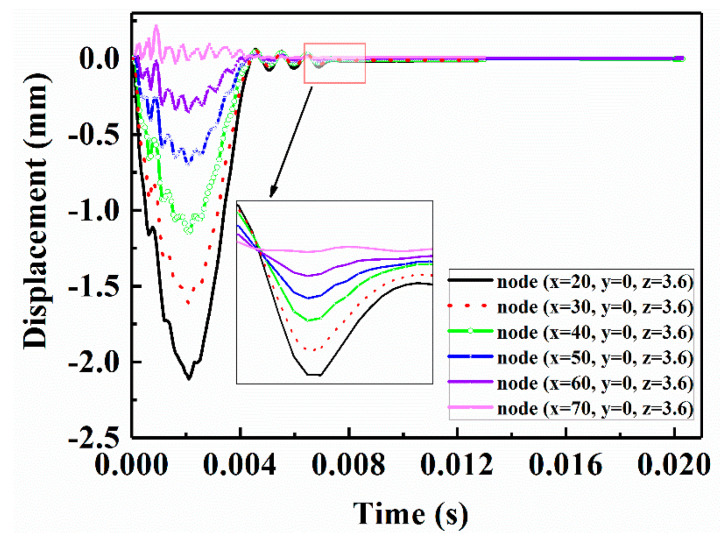
Displacement-time curves of six random nodes during the vibration damping step.

Figure 18 shows the displacement-time curves of six random nodes at different locations on the upper surface of the CFRP laminate that was repaired with a patch of radius 2.5 r at the end of the vibration damping step. To explain the effective influence of using the vibration damping step, the displacement-time vibration data in the U3 direction (Z-direction) from the start of the impact to the end of the vibration damping step were extracted. From the partially magnified figure in Figure 18, we can see that node x = 70, y = 0, z = 3.6 (the coordinate origin of the plate center) restores the equilibrium state first, whereas node x = 20, y = 0, z = 3.6 restores the equilibrium state last. That is, the closer the node is to the center position of the CFRP laminate, the more time is required to restore balance. The receiving point (x = 40, y = 0, z = 3.6) was selected for nonlinear Lamb wave detection in this study.

### 5.4. Influence of Patch Size on the Nonlinear Lamb Wave

**Figure 19:**
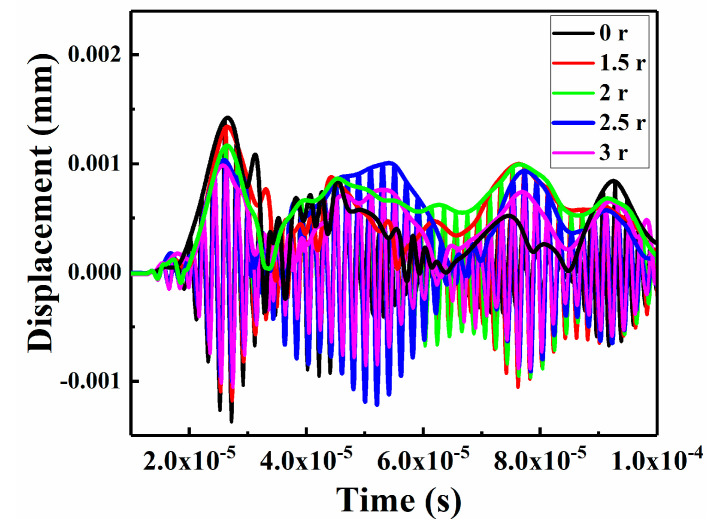
Predicted time-domain signal at the receiving point.

**Figure 20:**
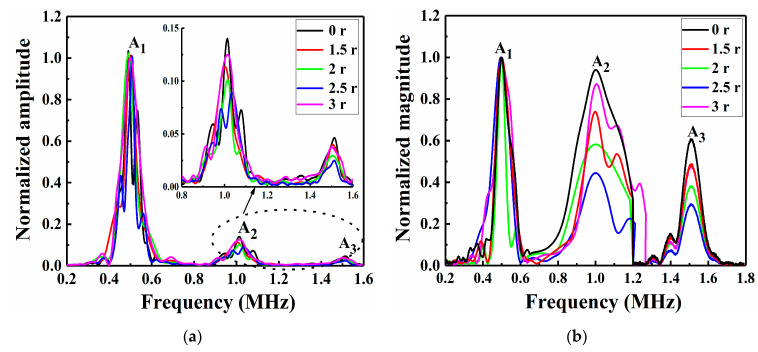
Frequency-domain responses of specimens repaired with patches of various sizes: (**a**) numerical and (**b**) experimental results.

The numerical time-domain signal curves of the CFRP laminates that were repaired with patches of various sizes are shown in Figure 19. With an increase in the patch size (first wave packet), the envelope displacement of the signal decreased, which might be attributed to a reduction in the internal Lamb wave propagation by the patch repairs. For a detailed study of higher harmonics, the numerical envelope frequency-domain signals were converted via FFT, as shown in Figure 20a. The experimental frequency-domain signal data, including the output frequency signals of A_1_, A_2_ and A_3_, were directly stored and outputted by the computer that was connected to the RAM-5000 SNAP system, as shown in Figure 20b. When A_1_ was uniformly normalized, it was found that increasing the patch size resulted in a trend of first decreasing and then increasing slowly in the second and third harmonic amplitudes. This is because, on the one hand, increasing the patch size is equivalent to increasing the bearing area, so it can improve the strength of the repaired CFRP laminate structure; on the other hand, increasing the patch size also increases the bonding area and reduces the peeling stress of the adhesive layer, which is beneficial to reducing the degumming phenomenon. Thus, with an increase in the patch size, the patch effectiveness increases, the impact damage decreases, and the nonlinear effect of NDT decreases. However, increasing the patch size has a critical value. If the patch size continued to increase beyond this point, the weight of the structure would be increased, which is of no use to the strength of the repaired structure, and if the bearing area of the patch is too large and close to the impact position, the impact damage would increase, the nonlinear effect of NDT would increase, and the patch-repair technique would become meaningless. Hence, when the patch radius reached 2.5 r, the corresponding nonlinear effect reached the minimum value, and the repair performance was relatively good. Additionally, the experimental results are consistent with the simulation results, which further verifies the effectiveness of the FE model.

**Figure 21:**
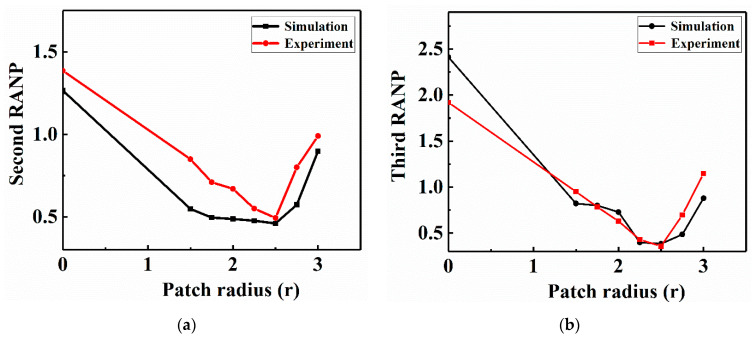
Numerical and experimental curves of the CFRP laminates repaired with patches of various sizes: (**a**) second RANP and (**b**) third RANP.

To quantitatively analyze the effects of LVI damage caused by the specimens repaired with patches of various sizes on the Lamb wave nonlinearities, the RANPs are used as a quantitative indicator of the medium nonlinearity. The RANPs reflect the degree of waveform distortion when ultrasonic waves pass through nonlinear materials. The larger the RANPs are, the more serious the internal damage of the repaired structure, and vice versa. Therefore, the second RANP and third RANP were utilized to evaluate the impact damage and measure the repair effectiveness of the repaired structure after impact. Therefore, the data without normalized A_1_, A_2_ and A_3_ in the FE simulation models and experiments were extracted. The second and third RANP values were obtained by solving Equations (2) and (3), respectively, as shown in Figure 21. The second and third RANP curves first decreased and then increased with increasing patch size. This is because the use of appropriate repair-patch design parameters can significantly improve the impact resistance of CFRP laminates containing holes and reduce the nonlinear Lamb wave propagation response, so the RANPs decrease. When the radius of the patch reached 2.5 r, both the second RANP and the third RANP were minimized. If the patch size reaches 2.5 r and then continues to increase beyond this radius, the RANPs of the patch-repair CFRP laminates fluctuate around the converged value. Additionally, with the increase in the patch size, the patch is closer to the impact position, the patch damage is more serious, the nonlinear effect of the NDT increases, and the RANPs increase. This result confirmed that the specimen that was repaired with the patch of radius 2.5 r exhibited better repair performance than the other specimens, which is consistent with the conclusion in reference [7] that the optimal impact resistance repair design parameter for repaired CFRP laminates is a circular patch of radius 2.5 r. Moreover, the trend of the simulation was essentially in accordance with that of the experiment, so the simulation results were verified by nonlinear Lamb wave detection experiments.

### 5.5. Selection of the Optimal Patch Design

The previous section discussed the effects of patches of various sizes on the CFRP laminate repair behaviors, and the specimen repaired with a patch of radius 2.5 r demonstrated a better impact resistance behavior than the other repaired specimens. Based on the obtained simulation results, a third-order polynomial interpolation method was established to predict the relationships between the patch radius *R*, absorbed energy *E_abs_*, delamination area *E_del_*, second RANP *E_sec_*, and third RANP *E_thi_*. The forms of the above polynomial interpolation variable equations are expressed as follows:

$$E_{abs}=0.0667R^{3}-0.0709R^{2}-1.0482R+6.8177$$

$$E_{del}=2.4819R^{3}+3.4911R^{2}-69.0436R+711.5956$$

$$E_{sec}=0.1483R^{3}-0.4555R^{2}-0.1012R+1.2631$$

$$E_{thi}=0.2084R^{3}-0.6462R^{2}-0.4688R+2.4064$$

**Figure 22:**
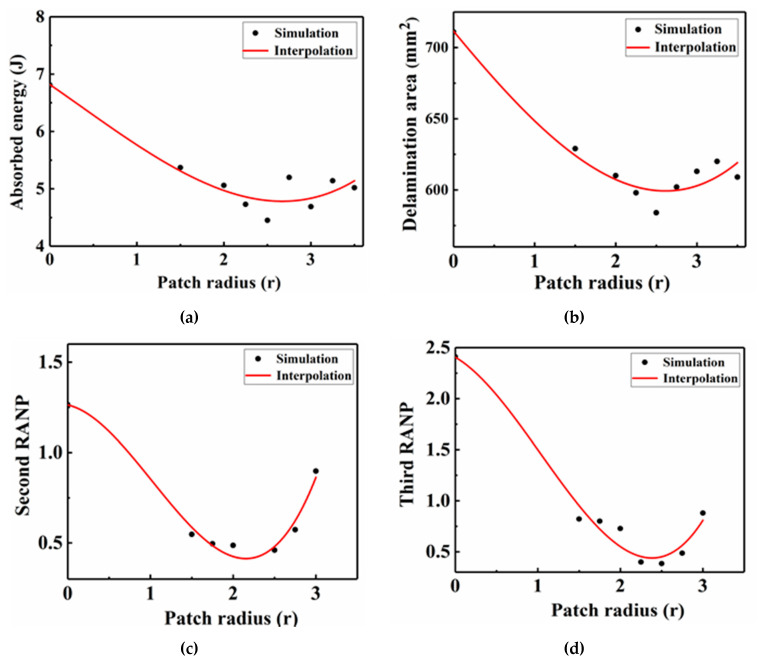
Interpolation relationships of the patch radius with the (**a**) absorbed energy, (**b**) delamination area, (**c**) second RANP and (**d**) third RANP.

Figure 22 shows the absorbed energy, delamination area, second RANP, and third RANP as functions of the patch radius, and the numerical simulation data of different patch sizes are represented by black dots. The minimum fitting curve values for the absorption energy, delamination area, second RANP, and third RANP correspond to patch sizes of approximately 2.69 r, 2.61 r, 2.35 r, and 2.38 r, respectively. Herein, we used a patch design with a circular geometry and a radius of approximately 2.5 r as the optimal patch. In addition, the absorbed energy, delamination area, second RANP, and third RANP showed consistent curve fitting trends, which indicates that they can be used as the main indexes to evaluate LVI damage.

## 6. Conclusions

An integrated numerical simulation procedure combined with LVI behavior and nonlinear Lamb wave detection has been established to predict the nonlinear Lamb wave propagation characteristics in damaged CFRP laminates that were repaired with patches of various sizes under LVI. This procedure provides an excellent research method to evaluate the interaction relationship between nonlinear Lamb waves and LVI damage. Herein, patch-repaired specimens—CFRP laminates that contain manufactured holes as existing damage—were subjected to drop-weight impact tests, and a RAM-5000 SNAP detection system was successfully employed to investigate the influences of patches of various sizes. The numerical predictions coincided well with the experimental results, which validated the correctness of the numerical simulation model. In addition, the necessity and feasibility of the vibration damping step were verified. The numerical simulation data of the impact force, absorbed energy, second RANP, and third RANP corroborated the experimental detection results. Based on the polynomial interpolation method, the absorbed energy, delamination surface areas, second RANP, and third RANP exhibited consistent curve fitting trends, which indicates that they can be used as the main indexes to evaluate LVI damage. The optimal patch design was selected, and excellent repair performance was obtained.

## Figures and Tables

**Table 1:** Mechanical material properties of the CFRP laminates and adhesive.

T300/7901		Adhesive	
*E*_1_/GPa	125.90	*Gt IC*/(N·mm^−1^)	0.52
*E*_2_, *E*_3_/GPa	11.30	*Gc IC*/(N·mm^−1^)	0.92
*G*_12_, *G*_13_/GPa	5.43	*σ*_n,max_/MPa	50
*G*_23_/GPa	3.97	*σ*_s,max_/MPa	94
*v*_12_, *v*_13_	0.30	*σ*_t,max_/MPa	94
*v*_23_	0.42	*K*_n_/(N·mm^−3^)	100,000
*X*_T_/MPa	2000	*K*_s_, *K*_t_/(N·mm^−3^)	100,000
*X*_C_/MPa	1100		
*Y*_T_, *Z*_T_/MPa	80		
*Y*_C_, *Z*_C_/MPa	280		
*S*/MPa	120		
*ρ*/kg·m^−3^	1478		

**Table 2:** Experimental and numerical delamination surface area results.

Label	Experiment (mm^2^)	FE Simulation (mm^2^)	Error (%)
0 r	700	711	1.57
2.5 r	559	584	4.47

**Table 3:** Experimental and simulation results of the relative acoustic nonlinearity parameters (RANPs).

Label	Method	A_1_	A_2_	A_3_	*β*′	δ′
0 r	Experiment	0.63	0.55	0.48	1.3857	1.9196
	FE simulation	0.1232	0.0192	0.00451	1.2650	2.4118
2.5 r	Experiment	0.90	0.40	0.26	0.4938	0.3567
	FE simulation	0.2432	0.02722	0.00553	0.4602	0.3844

## Data Availability

No new data were created or analyzed in this study. Data sharing is not applicable to this article.
